# Oxidative stress-induced Notch1 signaling promotes cardiogenic gene expression in mesenchymal stem cells

**DOI:** 10.1186/scrt190

**Published:** 2013-04-18

**Authors:** Archana V Boopathy, Karl D Pendergrass, Pao Lin Che, Young-Sup Yoon, Michael E Davis

**Affiliations:** 1 Wallace H. Coulter Department of Biomedical Engineering, Emory University and Georgia Institute of Technology, 101 Woodruff Circle, Suite 2001, Atlanta, GA 30322, USA; 2 Interdisciplinary BioEngineering Program, Georgia Institute of Technology, Atlanta, GA 30332, USA; 3 Parker H. Petit Institute for Bioengineering and Bioscience, Atlanta, GA 30332, USA; 4 Division of Cardiology, Emory University School of Medicine, Atlanta, GA 30332, USA

**Keywords:** Cardiac progenitor cells, Gene expression, Glucose oxidase, Hydrogen peroxide, Mesenchymal stem cells, Notch1

## Abstract

**Introduction:**

Administration of bone marrow-derived mesenchymal stem cells (MSCs) after myocardial infarction (MI) results in modest functional improvements. However; the effect of microenvironment changes after MI, such as elevated levels of oxidative stress on cardiogenic gene expression of MSCs, remains unclear.

**Methods:**

MSCs were isolated from the bone marrow of adult rats and treated for 1 week with H_2_O_2_ (0.1 to 100 μ*M*) or 48 hours with glucose oxidase (GOX; 0 to 5 mU/ml) to mimic long-term pulsed or short-term continuous levels of H_2_O_2_, respectively.

**Results:**

In 100 μ*M* H_2_O_2_ or 5 mU/ml GOX-treated MSCs, mRNA expression of selected endothelial genes (*Flt1, vWF, PECAM1*), and early cardiac marker (*nkx2-5, αMHC*) increased significantly, whereas early smooth muscle markers (*smooth muscle α-actin and sm22α*) and fibroblast marker *vimentin* decreased, as measured with real-time PCR. Interestingly, mRNA expression and activity of the cell-surface receptor Notch1 were significantly increased, as were its downstream targets, *Hes5* and *Hey1*. Co-treatment of MSCs with 100 μ*M* H_2_O_2_ and a γ-secretase inhibitor that prevents Notch signaling abrogated the increase in cardiac and endothelial genes, while augmenting the decrease in smooth muscle markers. Further, on GOX treatment, a significant increase in Wnt11, a downstream target of Notch1, was observed. Similar results were obtained with adult rat cardiac-derived progenitor cells.

**Conclusions:**

These data suggest that H_2_O_2_- or GOX-mediated oxidative stress upregulates Notch1 signaling, which promotes cardiogenic gene expression in adult stem/progenitor cells, possibly involving Wnt11. Modulating the balance between Notch activation and H_2_O_2_-mediated oxidative stress may lead to improved adult stem cell-based therapies for cardiac repair and regeneration.

## Introduction

Cardiovascular disease is the leading cause of global morbidity and mortality [1]. Among cardiovascular diseases, myocardial infarction (MI) leads to irreversible damage to the myocardium and progressive loss of function, eventually leading to heart failure. Although many therapies attempt to improve functionality of the heart, the only cure for heart failure is cardiac transplantation. Owing to low availability of donor hearts, newer treatments that restore tissue and function are greatly needed. Although studies have shown functional improvement on injection of various stem cells, the precise molecular mechanism responsible for the improvement is unclear. Accumulating lines of evidence indicate beneficial effects of bone-marrow-derived mesenchymal stem cells (MSCs) for treating MI in small- and large-animal studies [2]. Whereas MSCs are capable of differentiating into multiple lineages [3], on delivery to the heart after acute MI, cardiogenic (all cell types in the myocardium) differentiation was noted [4]. Additionally, several studies have shown improvements in cardiac function after MI either by endogenous bone marrow [5] or by augmenting this endogenous MSC response via injection of Granulocyte-macrophage colony-stimulating factor (GM-CSF) and other stem cell-mobilizing factors [6]. Finally, early human clinical trials demonstrated modest, yet significant, improvements in cardiac performance after MSC administration [7,8].

Extensive published studies demonstrate significant increases in reactive oxygen species (ROS) almost immediately after an acute MI. Elevated levels of ROS have deleterious effects on the cardiovascular system and are critical in the pathophysiology of heart failure. Potential sources of ROS in the myocardium include the NADPH oxidases of fibroblasts and infiltrating inflammatory cells, as well as the myocytes themselves [9]. Ischemic injury further elevates ROS production in these cells, which may influence differentiation of endogenous or implanted stem cells at the infarct site. Although effects of ROS on cardiac cell death, remodeling, and function are well studied, stem and progenitor cells that could be used for potential regeneration have both adaptive and maladaptive responses to oxidative stress. For example, acute bursts of ROS to embryonic stem cells (ESCs) in culture facilitate differentiation toward the cardiomyocyte phenotype, whereas prolonged exposure to H_2_O_2_ inhibits differentiation [10]. Although the effect of ROS on certain stem cell types is fairly established, the exact signaling pathways regulated by ROS, especially in the cardiogenic differentiation of stem cells, are under intensive investigation. Thus, pinpointing the exact signals modulated by ROS, leading to alterations in MSC differentiation, is of great therapeutic interest.

One of the major signaling pathways involved in stem cell differentiation is the Notch signaling pathway. Notch signaling is an evolutionarily conserved intercellular communication pathway that regulates diverse cellular processes, ranging from cell-fate decision, differentiation, and proliferation to apoptosis. Activation of the Notch receptor by adjacent cell surface-bound ligands of the Jagged and Delta family leads to proteolytic cleavage and nuclear translocation of the Notch intracellular domain (NICD) and subsequent transcriptional regulation of target genes, leading to maintenance of cells in an uncommitted state or induction of cell-type-specific differentiation [11]. Notch signaling promotes early cardiac development [12] and has also been identified to precede heart regeneration in zebrafish [13]. Further, certain mutations in Notch ligands or receptors are associated with embryonic lethality in mice [14]. Apart from regulating normal development and damage-induced repair, Notch signaling has also been found to promote cardiomyocyte survival [15]. Notch activation has been shown to promote cardiac gene expression in circulating endothelial progenitor cells [16], bone-marrow derived MSCs [17], and cardiac progenitors [18], while attenuating cardiac differentiation of embryonic stem cells [19].

The effects of Notch signaling on different cell types are well studied, but its regulation by oxidative stress is unknown. We therefore sought to determine the role of oxidative stress on cardiogenic gene expression in MSCs and whether Notch signaling plays a role in directing differentiation of MSCs in the setting of elevated local H_2_O_2_ levels after MI. Our data suggest that H_2_O_2_ or glucose oxidase-mediated oxidative stress promotes cardiogenic differentiation in adult stem/progenitor cells through upregulation of Notch1 signaling, possibly involving Wnt11.

## Materials and methods

### Mesenchymal stem cell isolation

Mesenchymal stem cells were obtained from the femur and tibia of adult male Sprague–Dawley rats with Percoll density gradient centrifugation and adherence to tissue-culture flasks. MSCs were used from passages 2 to 4 cultured on Minimal Essential Media alpha (MEMα; Hyclone, Logan, USA) supplemented with 20% fetal bovine serum (Hyclone), L-glutamine (Cellgro, Mediatech, USA), and 100 U penicillin-streptomycin (Invitrogen, Carlsbad, USA).

### Trilineage differentiation of MSCs

To determine the trilineage differentiation capacity, the MSCs were cultured in adipogenic (SR811D250; Amsbio, Abingdon, UK), osteogenic (SR417D250; Amsbio), or chondrogenic (SC00B5-2; Vitro Biopharma, Golden, USA) media for 21 days with media replenishment every 3 days. The MSCs were also cultured for 1 week ± 100 μ*M* H_2_O_2_. To demonstrate adipogenic differentiation, the cells were stained with 0.3% Oil Red O (O0625; Sigma, St. Louis, USA) in isopropanol for 30 minutes and rinsed with PBS. For osteogenic differentiation, the cells were stained with 1% alizarin red (500–4; RiccaChem, Arlington, USA) for 15 minutes. For chondrogenic differentiation, cells were stained with 0.5% toluidine blue O in PBS (198161-5G; Sigma). The stained cells were imaged under a phase-contrast microscope (Olympus, Pittsburgh, USA).

### Cardiac progenitor cell isolation

Cardiac progenitor cells (CPCs) were isolated from the hearts of adult male Sprague–Dawley rats by selection of cKit^+^ cells with anti-cKit antibody (H-300; Santa Cruz, Dallas, USA)-coated magnetic beads (Dynal, Carlsbad, USA), as previously described [20].

### Characterization of MSCs and CPCs

The surface expression of c-kit (H-300; Santa Cruz), CD45 (Invitrogen), CD34 (sc-7324; Santa Cruz), CD73 (551123; BD Pharmingen, San Jose, USA), CD90 (554898; BD Pharmingen, San Jose, USA), and CD105 (bs-0579R; Bioss, Denver, USA) in MSCs and expression of c-kit and the transcription factors nkx2-5 (sc-14033; Santa Cruz), and gata4 (sc-9053; SantaCruz) in CPCs was determined with flow analysis by using an FACSCalibur (Becton Dickinson, New Jersey, USA). The isotypes of each antibody served as the negative control.

### Induction of oxidative stress in MSCs and CPCs

To induce acute oxidative stress, MSCs or CPCs were cultured in serum-free media with Insulin/Transferrin/Selenium (ITS) containing H_2_O_2_ (0 to 100 μ*M*; Fisher Scientific, New Hampshire, USA) for 1 week, or glucose oxidase (0 to 5 mU/ml; Sigma) for 48 hours. Media was replenished every day with fresh media with or without H_2_O_2_. MSC and CPC growth media contain 5.5 m*M* and 10 m*M* glucose, respectively, and the addition of GOX results in continuous generation of H_2_O_2_.

### Gene expression

Total RNA was isolated by using the QIA RNeasy kit (Qiagen, Valencia, USA) as per maunfacturer’s instructions. First-strand cDNA was synthesized as described [21]. Quantitative real-time PCR was performed on a StepOne Plus real-time PCR system (Applied Biosystems, Carlsbad, USA) by using specific primers for the cardiogenic and Notch1-related genes (see Additional file 1, Table S1). Gene-expression data were normalized to GAPDH in the H_2_O_2_-treated MSCs and to 18S in the GOX-treated MSCs. GAPDH is a gene involved in glucose metabolism and, as addition of glucose oxidase (GOX) alters the glucose levels in the media, 18S and not GAPDH was used as the housekeeping gene for studies involving GOX.

### Protein expression

MSCs were treated with or without 5 mU/ml GOX for 48 hours. The protein expression of α-MHC (ab50967; Abcam, Cambridge, UK), Flt1 (ab32152; Abcam, Cambridge, UK), and smooth muscle α-actin (SAB250093; Sigma) was determined with flow analysis by using an FACSCalibur (Becton Dickinson). Primary antibodies were used at 1:300, and appropriate secondary antibodies were used at 1:500 with isotype controls.

### Measurement of Notch intracellular domain

Total protein was isolated as described [21]. Equal amounts of protein were loaded on 4% to 15% SDS-PAGE gradient gel (Bio-Rad, Berkeley, USA). After transfer, the nitrocellulose membrane was probed with anti-Notch intracellular domain (Cell Signaling, Beverly, USA) antibody. A horseradish peroxidase-conjugated goat anti-rabbit secondary antibody was used (Bio-Rad), and chemiluminescent signals were obtained on a Kodak Imager Station 4000 MM Pro (Carestream Molecular Imaging, Rochester, USA). NICD protein levels were normalized to GAPDH (Santa Cruz).

### Chemical inhibition of Notch signaling

MSCs were treated with a γ-secretase inhibitor IX DAPT (*N*-[*N*-(3,5-difluorophenacetyl-L-alanyl)]-*S*-phenylglycine *t*-butyl ester; Calbiochem, Billerica, USA, 10 μ*M*) every day for 1 week to inhibit Notch1 activation in MSCs ± H_2_O_2_. On day 7, RNA and protein were harvested for subsequent qRT-PCR and Western blotting, respectively.

### siRNA-mediated knockdown of Notch1

To determine the optimal transfection reagent, MSCs were transfected with 100 n*M* mock siRNA labeled with Cy3 for 48 hours by using oligofectamine (Life Technologies, Carlsbad, USA), HiPerfect (Qiagen), or Lipofectamine RNAimax (Life Technologies), according to the manufacturer’s instructions. The transfection efficiency was determined with flow analysis (FACSCalibur; Becton Dickinson) and fluorescent microscopy (Nikon). To knockdown Notch1 expression in MSCs, the cells were transfected with 25 n*M* either QIAgen Allstar Negative control siRNA (siNC) or QIA siNotch1 (S101920730) with oligofectamine (Life Technologies), according to the manufacturer’s instructions. After 48 hours, RNA and protein were harvested for subsequent qRT-PCR and Western blotting, respectively.

### Gene-expression analysis with PCR array

Based on the manufacturer’s instructions, the Qiagen Rat Notch PCR Array PARN-059A was used to analyze gene expression in MSCs treated with or without 5 mU/ml GOX.

### Statistical analysis

All data are expressed as mean ± SEM. To determine significance, either an analysis of variance (ANOVA) was done followed by the appropriate *post hoc* test, or a Student *t* test was performed by using GraphPad Prism5. A *P* value <0.05 was considered significant.

## Results

### Characterization of mesenchymal stem cells

**Figure 1:**
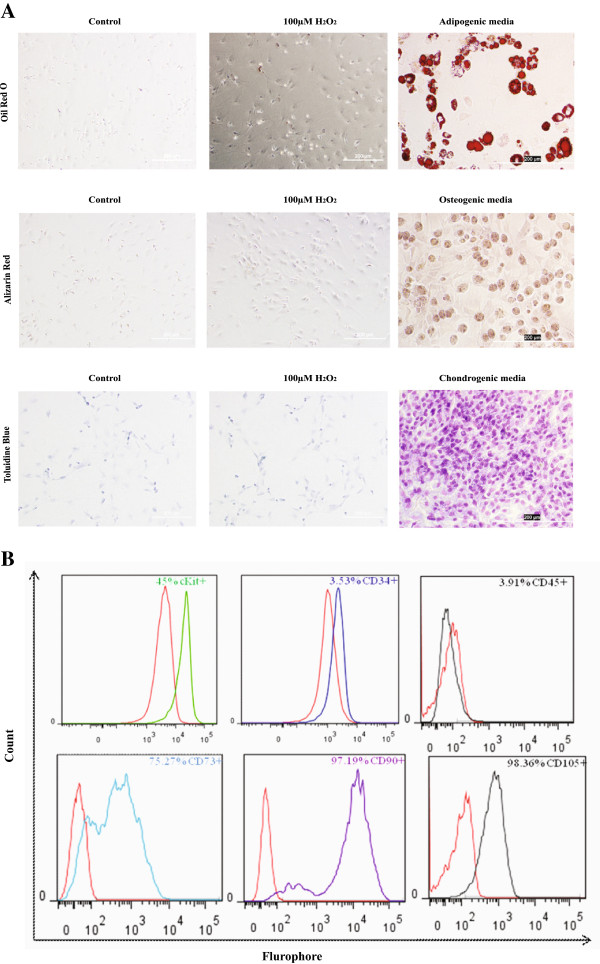
**Characterization of mesenchymal stem cells. (A) **The trilineage differentiation potential of the mesenchymal stem cells (MSCs) cultured on adipogenic, osteogenic, and chondrogenic media or ±100 μ*M *H_2_O_2 _was assessed with oil red O, alizarin red, and toluidine blue staining, respectively. **(B) **The MSCs isolated from rat bone marrow were culture expanded, and the percentage of positive cells expressing cKit, CD34, CD45, CD73, CD90, and CD105 was determined with flow analysis. Scale bar, 200 μm.

Mesenchymal stem cells (MSCs) had a spindle-shaped, fibroblast-like morphology and expressed common mesenchymal cell-surface markers, c-Kit, CD73, CD90, and CD105, with low expression of the hematopoietic markers CD45 and CD34 (Figure 1B). To determine the multipotent trilineage differentiation capacity, the MSCs were cultured for 21 days in media that promote differentiation into adipogenic, osteogenic, and chondrogenic lineages. As shown in Figure 1A, the MSCs differentiated into the three lineages, as demonstrated by staining for oil red O, alizarin red, and toluidine blue, respectively. Moreover, treatment with 100 μ*M* H_2_O_2_ for 1 week did not induce trilineage differentiation, indicating that the MSCs are multipotent, but H_2_O_2_ treatment does not promote differentiation into adipogenic, osteogenic, or chondrogenic lineages.

### H_2_O_2_ or glucose oxidase treatment increases early endothelial gene expression in MSCs

**Figure 2:**
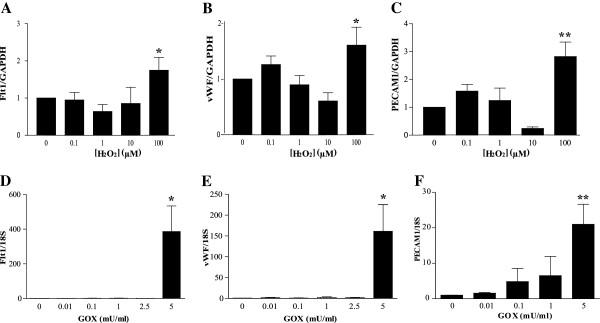
**Oxidative stress promotes endothelial gene expression in mesenchymal stem cells (MSCs). **MSCs were treated with either H_2_O_2 _for 1 week (0 to 100 μ*M*; **A **through **C**) or GOX for 48 hours (0 to 5 mU/ml; **D **through **F**). Expression of the endothelial markers Flt1 (**A**, **D**), vWF (**B**, **E**), and PECAM1 (**C**, **F**) was determined with qRT-PCR. Values are expressed as mean ± SEM after normalizing gene expression to GAPDH (**A **through **C**) or 18S (**D **through **F**) expression. For **A **through **C**, *n *≥ 4, and **D **through **F**, *n* ≥ 6. *P < 0.05 and **P < 0.01 when compared with control (0 μ*M *H_2_O_2 _or 0 mU/ml GOX) by one-way ANOVA followed by Dunnett posttest.

To determine whether H_2_O_2_ or glucose oxidase (GOX)-mediated oxidative stress regulates endothelial gene expression, bone marrow-derived MSCs were cultured in media containing hydrogen peroxide (H_2_O_2_; 0 to 100 μ*M*) for 1 week or glucose oxidase (GOX; 0 to 5 mU/ml) for 48 hours. The mRNA expression of VEGF receptor *Flt1, vWF, and PECAM1* after exposure to H_2_O_2_ or GOX was quantified with qRT-PCR. Although no effect was due to lower levels of H_2_O_2_ (0.1 to 10 μ*M*), high levels (100 μ*M*) significantly increased the expression of *Flt1* by twofold (*P* < 0.05; Figure 2A), *vWF* by almost 2-fold (*P* < 0.05; Figure 2B), and *PECAM1* by 3-fold (*P* < 0.01; Figure 2C). In MSCs treated with GOX for 48 hours, no change in gene expression was seen at the lower doses (0.01 to 2.5 mU/ml). However, at 5 mU/ml, a significant increase in expression of *Flt1* by 400-fold (*P* < 0.05; Figure 2D), *vWF* by 150-fold (*P* < 0.05; Figure 2E), and *PECAM1* by 20-fold (*P* < 0.01; Figure 2F) was observed.

### H_2_O_2_ or glucose oxidase treatment increases early cardiac gene expression in MSCs

**Figure 3:**
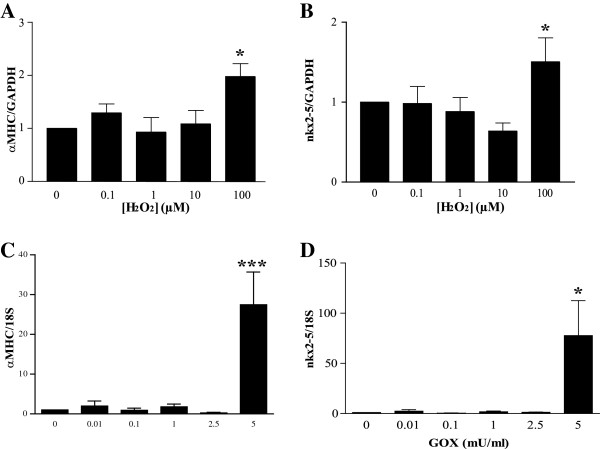
**Oxidative stress regulates cardiac gene expression in mesenchymal stem cells (MSCs). **MSCs were treated with either H_2_O_2 _for 1 week (0 to 100 μ*M*; **A**, **B**) or GOX for 48 hours (0 to 5 mU/ml; **C**, **D**). Expression of the cardiac markers αMHC (**A**, **C**) and nkx2-5 (**B**, **D**) was determined with qRT-PCR. Values are expressed as mean ± SEM after normalizing gene expression to GAPDH (**A**, **B**) or 18S (**C**, **D**) expression. For **A **and **B**, *n *≥ 4; **C** and **D**, *n *≥ 5. *P < 0.05 and ***P < 0.001 when compared with control (0 μ*M *H_2_O_2 _or 0 mU/ml GOX) with one-way ANOVA followed by Dunnett posttest.

To determine whether H_2_O_2_ or GOX-mediated oxidative stress regulates cardiac gene expression, we measured levels of early cardiac markers *nkx2-5* and α-myosin heavy chain (*αMHC*). Whereas treatment of MSCs with low levels of H_2_O_2_ (0.1 to 10 μ*M*) had no effect on *αMHC* levels, treatment with 100 μ*M* H_2_O_2_ significantly increased expression by 2-fold (*P* < 0.05; Figure 3A) compared with time-matched, untreated controls. To confirm with another early cardiac marker, *nkx2-5*, gene expression was measured. As shown in Figure 3B, the expression of *nkx2-5* increased by nearly 2-fold (*P* < 0.05) in 100 μ*M* H_2_O_2_-treated MSCs. In MSCs treated with GOX for 48 hours, no change in gene expression was seen at the lower doses (0.01 to 2.5 mU/ml). In MSCs treated with 5 mU/ml GOX, a 30-fold increase in expression of αMHC (*P* < 0.001; Figure 3C) was observed, along with a 75-fold increase in nkx2-5 expression (*P* < 0.05; Figure 3D).

### H_2_O_2_ or glucose oxidase treatment negatively modulates smooth muscle and fibroblast gene expression in MSCs

**Figure 4:**
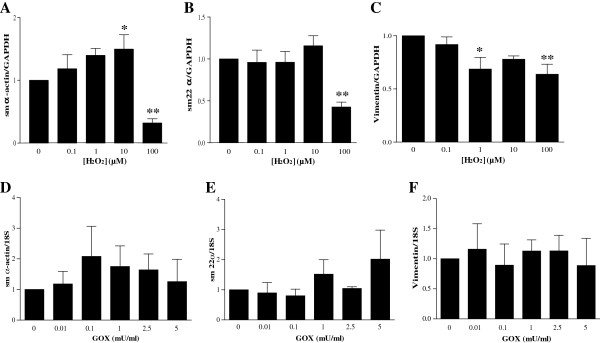
**Oxidative stress differentially modulates smooth muscle and fibroblast gene expression in mesenchymal stem cells (MSCs). **MSCs were treated with either H_2_O_2 _for 1 week (0 to 100 μ*M*; **A **through **C**) or GOX for 48 hours (0 to 5 mU/ml; **D **through **F**). Expression of the smooth muscle markers sm α-actin (**A**,**D**), sm22α (**B**,**E**), and the fibroblast marker vimentin (**C**, **F**) was determined with qRT-PCR. Values are expressed as mean ± SEM after normalizing gene expression to GAPDH expression. For **A **through **C**, *n *≥ 3; and **D**-**F**, *n *= 6. *P < 0.05 and **P < 0.01 when compared with control (0 μ*M *H_2_O_2 _or 0 mU/ml GOX) by one-way ANOVA followed by the Dunnett posttest.

To identify whether H_2_O_2_ regulated expression of smooth muscle markers, the levels of the early smooth muscle markers, smooth muscle α-actin (*sm α-actin*) and the calponin-related protein *sm22α* were examined. Treatment with 100 μ*M* H_2_O_2_ significantly decreased expression of *sm α-actin* by 3-fold (*P* < 0.01; Figure 4A) and *sm22α* expression by 2-fold (*P* < 0.01; Figure 4B). Similarly, the expression of vimentin, an intermediate-filament protein characteristic of fibroblasts, was significantly decreased by 1.5-fold in both 1 μ*M* (*P* < 0.05) and 100 μ*M* H_2_O_2_-treated MSCs (*P* < 0.01), as shown in Figure 4C. No change in expression of *sm α-actin, sm22α, and vimentin* was observed in MSCs treated with GOX (0 to 5 mU/ml), as in Figure 4D through F.

### H_2_O_2_ or glucose oxidase treatment induces Notch1 signaling

**Figure 5:**
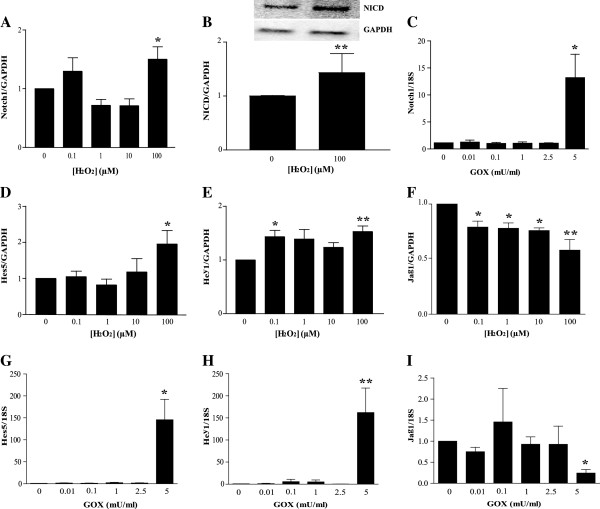
**Oxidative stress increases expression of Notch1 and its downstream targets.** Mesenchymal stem cells (MSCs) were treated with either H_2_O_2 _for 1 week (0 to 100 μ*M*; **A**, **B**, **D, E, F**) or GOX for 48 hours (0 to 5 mU/ml; **C**,**G** through **I**). The mRNA expression levels of Notch1 (**A**, **C**), Hes5 (**D**, **G**), Hey1 (**E**, **H**), and the ligand Jagged1 (**F**, **I**) was determined with qRT-PCR. (**B**) Quantification of expression of activated Notch1 (NICD) with representative Western blot image. Values are expressed as mean ± SEM after normalizing gene expression and Western blots to GAPDH (**A**,**B**,**D**,**E**,**F**) or 18S (**C**,**G**, **H**,**I**) expression and protein, respectively. For **A**, **B**, and **D **through **F**, *n *≥ 4; **C** and **G** through **I**, *n *≥ 6; *P < 0.05 and **P < 0.01 when compared with control (0 μ*M *H_2_O_2 _or 0 mU/ml GOX) by one-way ANOVA followed by the Dunnett posttest and Student unpaired *t *test for **B***.*

As the Notch signaling pathway has been shown to be critical for cardiovascular development and differentiation, we analyzed the expression levels of the cell-surface receptor *Notch1* and its ligand *Jagged1 (Jag1)* in H_2_O_2_- and GOX-treated MSCs. High levels of H_2_O_2_ (100 μ*M*) augmented Notch1 gene expression by 1.5-fold after 1 week and significantly increased Notch intracellular domain (NICD) cleavage (*P* < 0.05 for gene, *P* < 0.01 for NICD cleavage, Figure 5A and B), while decreasing *Jagged1 (Jag1)* expression by about 1.5-fold (*P* < 0.05, Figure 5F). Moreover, the expression of the downstream targets of Notch1, *Hes5*, was increased by up to twofold in MSCs treated with 100 μ*M* H_2_O_2_ (*P* < 0.05, Figure 5D) and *Hey1* by 1.5-fold (*P* < 0.01; Figure 5E). Although no change in gene expression was observed in MSCs treated with 0 to 2.5 mU/ml GOX, a significant increase in expression of *Notch1* (15-fold; *P* < 0.05; Figure 5C), *Hes5* (150-fold; *P* < 0.05; Figure 5G), *Hey1* (150-fold; *P* < 0.01; Figure 5H), and a significant decrease in expression of Jag1 (*P* < 0.05; Figure 5I) was observed in MSCs treated with 5 mU/ml GOX.

### mRNA analysis of Notch1-related genes regulated by GOX

**Figure 6:**
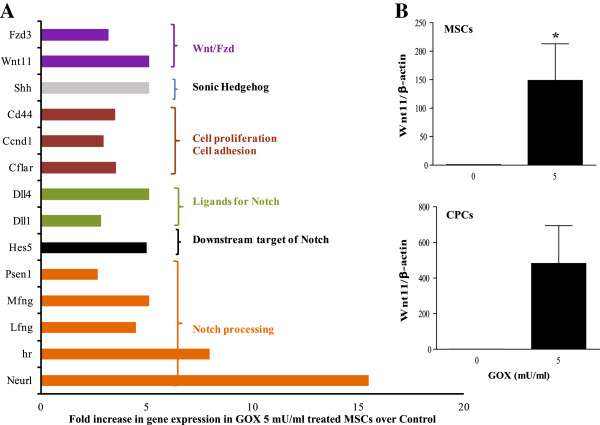
**Polymerase chain reaction (PCR) array analysis of Notch1-related genes. **Gene expression of Notch1-related genes in MSCs treated with GOX (0 or 5 mU/ml) was analyzed by using a PCR array. **(A) **Fold increase in gene expression in 5 mU/ml GOX-treated MSCs over control cells was normalized to β-actin. **(B)** Validation of increase in Wnt11 expression on 5 mU/ml GOX treatment in MSCs (top, *P *< 0.05) and CPCs (bottom, *P *= 0.08).

To determine the Notch1-related genes that were regulated in MSCs treated with 5 mU/ml of GOX, a PCR array was performed. The data were analyzed and grouped based on known associated function of the gene. As shown in Figure 6A, treatment of MSCs with 5 mU/ml of GOX resulted in an increase in expression of (a) *Wnt11* (fivefold) and its receptor *Fzd3* (3-fold), (b) genes involved in cell adhesion and proliferation (*Cd44, Ccnd1, Cflar*; about 3-fold), (c) ligands for Notch1 (*Dll1*, *Dll4*; 3-5 fold), (d) components of γ-secretase complex involved in Notch1 processing (*Psen1, Mfng, Lfng, hr, Neurl*; 3 to 15-fold); and (e) downstream target of Notch1 (*Hes5*; fivefold). As *Wnt11* is a known downstream target of Notch1, the expression of *Wnt11* was validated in MSCs treated with GOX (0 or 5 mU/ml). As shown in Figure 6B, a significant increase in Wnt11 expression was observed in MSCs treated with 5 mU/ml GOX. The results were also validated by using cardiac progenitor cells, in which a similar significant increase was observed (Figure 6B, bottom).

### H_2_O_2_ and glucose oxidase regulate cardiogenic gene expression in MSCs through Notch1

**Figure 7:**
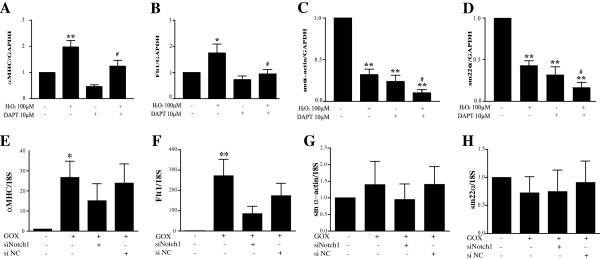
**H**_**2**_**O**_**2 **_**and glucose oxidase (GOX) regulate cardiogenic gene expression in mesenchymal stem cells (MSCs) in both Notch1-dependent and -independent manners.** MSCs were treated with 100 μ*M *H_2_O_2 _± Notch1 inhibitor DAPT (**A **through **D**) or with 5 mU/ml GOX ± siNotch1 (**E** through **H**). Expression of the cardiac marker αMHC (**A**, **E**), endothelial marker Flt1 (**B**,**F**), and the smooth muscle markers sm α-actin (**C**, **G**) and sm22α (**D**, **H**) were determined with qRT-PCR. Values are expressed as mean ± SEM after normalizing gene expression to GAPDH (**A**-**D**) or 18S (**E**-**H**) expression. *n* ≥ 4, *P < 0.05 and **P < 0.01 compared with control (0 μ*M *H_2_O_2_), or #*P *< 0.05 compared with 100 μ*M *H_2_O_2 _by one-way ANOVA followed by Bonferroni posttest.

To determine the link between increased Notch1 gene expression and activity by H_2_O_2_ treatment and cardiogenic gene regulation, MSCs were treated with a γ-secretase inhibitor IX DAPT (10 μ*M*) for 1 week with or without 100 μ*M* H_2_O_2_. γ-Secretase is required for the release of the active Notch1 intracellular domain (NICD) [22], and thus its inhibition blocks Notch1 activation. To demonstrate that DAPT inhibits Notch1 activation, MSCs were treated with DAPT (10 μ*M*) for 1 week with or without 100 μ*M* H_2_O_2_, and protein levels of NICD were measured with Western blotting. As shown in Additional file 1, Figure S1, DAPT decreases NICD expression in the presence of 100 μ*M* H_2_O_2_. Inhibition of Notch1 signaling by DAPT for 1 week showed a small but nonsignificant decrease on basal αMHC expression, but prevented the increase seen in 100 μ*M* H_2_O_2_-treated cells (*P* < 0.05 versus H_2_O_2_ alone; Figure 7A). A similar effect of DAPT was observed in the expression pattern of the endothelial marker *Flt1*, in which Notch1 inhibition prevented the increase in *Flt1* expression by 100 μ*M* H_2_O_2_ (*P* < 0.05 versus H_2_O_2_ alone; Figure 7B). Interestingly, inhibition of basal Notch1 signaling by DAPT decreased expression of *sm α-actin* by 4-fold (*P* < 0.001, Figure 7C) and *sm22α* by 3-fold (*P* < 0.001, Figure 7D). This decrease was further augmented by the combined treatment with 100 μ*M* H_2_O_2_ and DAPT with a 10-fold and 6-fold decrease in expression of *sm α-actin* and *sm22α*, respectively (*P* < 0.001 versus H_2_O_2_ alone, Figure 7C and D).

To determine whether GOX mediated changes in cardiogenic gene expression through Notch1 signaling, MSCs were treated with 5 mU/ml GOX in the presence of a negative control siRNA (siNC) or siNotch1 for 48 hours. MSCs were transfected with siRNA by using oligofectamine, as it resulted in the highest transfection efficiency when compared with other commercially available reagents (Additional file 1, Figure S2A). As shown in Additional file 1, Figure S2B, siNotch1 significantly decreased mRNA expression of Notch1 (*P* < 0.05) when compared with untreated or siNC-treated MSCs. Further, siNotch1 decreased the GOX-induced increase in Notch1 expression comparable to untreated cells, whereas the siNC had no effect (Additional file 1, Figure S3A and B). As shown in Figure 7E and F, addition of siNotch1 showed a trend toward decreasing GOX-induced increase in expression of cardiac *αMHC and Flt1* with no significant effect of siNC. The expression of the smooth muscle markers (*sm α-actin* and *sm22α*) was unaltered by addition of GOX in presence or absence of siNC or siNotch1 (Figure 7G, H).

### H_2_O_2_ and glucose oxidase regulate cardiogenic protein expression

**Figure 8:**
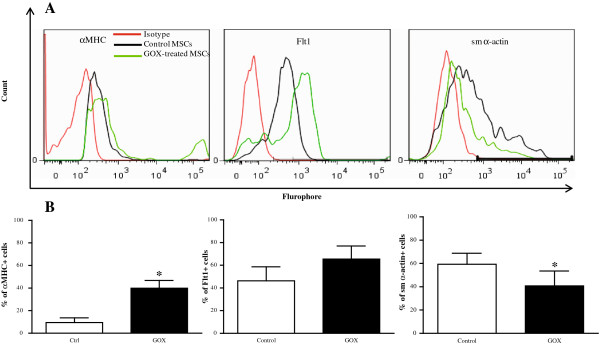
**Quantification of glucose oxidase (GOX)-mediated cardiogenic protein expression in mesenchymal stem cells (MSCs). **MSCs were treated with GOX (5 mU/ml) for 48 hours. Expression of the cardiac proteins (αMHC, Flt1, and sm α-actin) was determined with flow cytometry (**A**) and quantified in **B**. Values are expressed as mean ± SEM with *n *≥ 3. *P < 0.05 versus control with the Student paired *t *test.

To verify whether the changes in cardiogenic gene expression translated to changes in protein expression, levels of the cardiac marker *αMHC*, endothelial marker *Flt1, and smooth muscle α-actin* were determined with flow analysis (Figure 8A). As shown in the grouped data in Figure 8B, GOX induced a 4-fold increase in cardiac *αMHC*-expressing cells (left; *P* < 0.05), a 1.4-fold increase in Flt1-expressing cells (middle; *P* = 0.07), and a 1.5-fold decrease in sm α-actin-expressing cells (right; *P* < 0.05).

### Oxidative stress promotes cardiogenic gene expression in cardiac progenitor cells

**Figure 9:**
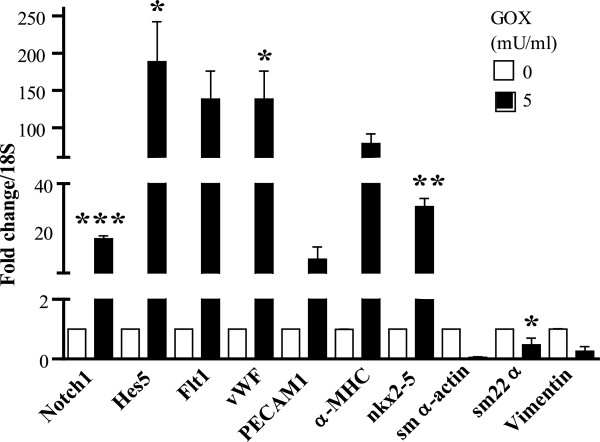
**Oxidative stress regulates cardiogenic gene expression in cardiac progenitor cells.** Cardiogenic gene expression in cardiac progenitor cells treated with GOX (0 or 5 mU/ml) was determined with qRT-PCR. An increase in expression of Notch1 (*P* < 0.001), Hes5 (*P *< 0.05), flt1 (*P *= 0.08), vWF (*P* < 0.05), nkx2-5 (*P *< 0.01), and sm22α (*P *< 0.05) was observed. Values are expressed as mean ± SEM after normalizing gene expression to 18S expression: *n *= 3, *P < 0.05, **P < 0.01, ***P < 0.001 compared with control (0 mU/ml GOX) with the Student unpaired *t *test.

To determine whether the effects of oxidative stress mediated by H_2_O_2_ are specific for MSCs, we subjected adult cardiac progenitor cells to GOX treatment. Interestingly, an increase in expression of *Notch1* by 15-fold (*P* < 0.001) and *Hes5* by 175-fold (*P* < 0.05), along with changes in cardiogenic gene expression similar to the response of MSCs, was observed (Figure 9).

## Discussion

Previous studies showed that transplantation of MSCs in the heart after MI leads to small but significant functional improvements [5]. Understanding the molecular mechanisms by which MSCs promote cardiac function, especially in the oxidative microenvironment after MI, will greatly aid in improving efficacy of stem cell-based therapies. After myocardial infarction, elevated levels of ROS have been found at the infarct site [10], suggesting that ROS such as H_2_O_2_ might influence the differentiation and function of implanted MSCs. As substantial amounts of ROS have been found in the area at risk after MI [23], and as ROS have been identified to play a critical role in the differentiation of other stem cell types [24,25], we chose to study the effect of H_2_O_2_ on MSC differentiation *in vitro*.

Here we showed that MSCs that were subjected to pulses of pathophysiologic levels of H_2_O_2_ for 1 week or continuous H_2_O_2_ produced by oxidation of glucose in the extracellular media by GOX for 48 hours increased the expression of early cardiac and endothelial genes with decreased expression of early smooth muscle genes.

Although only a twofold increase in cardiac markers is observed with H_2_O_2_ treatment, addition of GOX results in a more robust increase of 30-fold for *αMHC* and 75-fold for *nkx2-5*, which are comparable to neonatal rat ventricular cardiomyocytes, with 40-fold and 100-fold higher expression of *αMHC* and *nkx2-5*, respectively, when compared with untreated MSCs. These results were in agreement with those reported for human embryonic stem cells (ESCs) [25]. Similar results were obtained when adult heart-resident cardiac progenitor cells (CPCs) were treated with GOX. These data demonstrate redox-sensitive alteration in cardiogenic gene expression in MSCs and CPCs.

Our results also demonstrate that only high levels of exogenous H_2_O_2_ (100 μ*M*) and high concentrations of GOX (5 mU/ml) were able to regulate expression of Notch1 and cardiogenic genes. We believe this very narrow threshold effect may be due to a combination of factors, such as presence of basal H_2_O_2_ and constitutive expression of antioxidant enzymes by cells. Unpublished data from our laboratory demonstrate basal H_2_O_2_ levels of 1 μ*M* in cultured stem cells, as measured with electron paramagnetic spin resonance. Recent reports demonstrate that many stem cells, including MSCs, contain higher levels of antioxidants [26,27]. We measured H_2_O_2_ levels in stem cells after addition of 100 μ*M* H_2_O_2_ and found that the concentration reduced to 12 μ*M*, within an hour, indicating rapid scavenging of exogenous oxidants.

Finally, recent data from human MSCs determined higher levels of catalase and glutathione peroxidase, with no changes in superoxide dismutase compared with other stem cells and fully differentiated cells [27]. In that study, a threshold response with human MSCs demonstrated almost 80% survival at 4 m*M* H_2_O_2_, decreasing to <10% at 8 m*M*. Our studies indicate robust survival at 100 μ*M* H_2_O_2_ and 5 mU/ml GOX with cellular responses, but showed higher concentrations to be potentially cytotoxic. Taken together, these data demonstrate that many cells, especially stem and progenitor, have threshold responses with small windows of dose-responses.

Next, we investigated whether H_2_O_2_ regulates any signaling pathway involved in stem cell differentiation. One of the signaling pathways that greatly influence stem cell differentiation is the Notch signaling pathway [28]. Therefore, we investigated whether interplay existed between H_2_O_2_ and Notch1 signaling pathways. Interestingly, the mRNA level of *Notch1* as well as proteolytic cleavage of the Notch1 intracellular domain (NICD) was upregulated by treatment with 100 μ*M* either H_2_O_2_ or GOX, suggesting that high levels of H_2_O_2_ affect expression of both the mRNA and protein activity of Notch1. Although only a 1.5-fold increase in mRNA and twofold increase in NICD protein is observed, reports suggest that very small changes in Notch1 activation are sufficient to induce Notch1 signaling [11]. Furthermore, this increase in Notch1 also significantly increased mRNA expression of downstream targets of Notch1. As MSCs were pulsed with H_2_O_2_ for 1 week, discontinuous oxidative stress resulted in small fold changes in cardiac gene expression. Although these changes may not represent true differentiation, they suggest that H_2_O_2_ levels influence cardiac gene expression in MSCs.

Although upregulation of cardiac and endothelial genes by Notch1 signaling may appear to be counterintuitive, given the role of Notch signaling in suppressing cardiomyogenesis in ESCs [19], upregulation of *nkx2-5* and *vWF* is consistent with reports indicating involvement of Notch1 signaling in regulating these genes in cardiac progenitor cells and bone marrow stromal cells, respectively [18,29]. Our observation that treatment with 100 μ*M* H_2_O_2_ or 5 mU/ml GOX decreased expression of the Notch1 ligand *Jagged1* in MSCs is consistent with previous reports of an inverse relation between expression levels of Notch1 and Jagged 1 in other cell types [30].

As we observed changes in cardiogenic gene expression at the mRNA level, we sought to determine whether these changes translated correspondingly at the protein level. Flow analysis of GOX-treated MSCs indicated that a small number of MSCs have high expression of *αMHC*, along with increased *Flt1* expression and decreased *sm α-actin* expression. These results suggest that GOX treatment increases the frequency *αMHC*- and *Flt1*-positive cells while decreasing *sm α-actin*-positive cells.

The mechanism of upregulation of Notch1 activation by H_2_O_2_ may be due to activation of enzymes involved in Notch1 cleavage and processing. It is possible that H_2_O_2_ may increase Notch1 activation via γ-secretase activation, as H_2_O_2_-mediated increase in γ-secretase activation has been demonstrated in the pathogenesis of Alzheimer disease [31]. Pharmacologic inhibition of γ-secretase activity by using DAPT inhibits Notch1 activation in different stem cells [29,32]. Therefore, to determine whether H_2_O_2_ regulates cardiogenic gene expression in MSCs through Notch1 signaling, we blocked Notch1 activation daily by using DAPT and analyzed expression of the different cardiogenic markers in the presence and absence of 100 μ*M* H_2_O_2_. Among the markers analyzed, the increase in expression of the high-affinity VEGF receptor *Flt1* and the cardiac marker *αMHC* observed with 100 μ*M* H_2_O_2_ was abrogated by co-treatment with DAPT, indicating that H_2_O_2_ regulates *Flt1* and *αMHC* expression through Notch1 signaling. We attempted knockdown of Notch1 by using siRNA; however, this was not successful, as siRNAs that significantly reduced Notch1 gene expression greatly reduced cell survival over the 1-week period of treatment.

To determine whether glucose oxidase (GOX) mediated acute changes in cardiogenic gene expression through Notch1 signaling, MSCs were transfected with siNotch1 along with GOX for 48 hours. Treatment with siNotch1 showed a strong trend toward decreasing the GOX-mediated increase in *αMHC* and *Flt1*, whereas no effect was observed on smooth muscle gene expression by addition of GOX ± siNotch1. As *ADAM17* is involved in Notch1 processing, MSCs were pretreated with an *ADAM17* inhibitor. No effect was observed on H_2_O_2_-induced gene expression, nor was the expression or activity of *ADAM17* altered by H_2_O_2_ treatment, suggesting the importance of the γ-secretase component of this pathway (Additional file 1, Figure S4). Further, treatment with GOX increased expression of enzymes involved in processing of Notch1 and Jagged1, such as *Mfng*, *Lfng,* and *Neurl,* indicating that H_2_O_2_ influences both notch1 cleavage and processing enzymes.

Expression of smooth muscle markers decreased significantly on treatment with 100 μ*M* H_2_O_2_. Inhibition of Notch1 also decreased basal expression of smooth muscle markers, in keeping with prior findings [33]. Interestingly, co-treatment of MSCs with H_2_O_2_ and DAPT resulted in a further decrease in smooth muscle markers. This indicates that H_2_O_2_ decreases smooth muscle gene expression through a parallel pathway, and that activation of Notch serves as a compensatory mechanism to stabilize smooth muscle gene expression.

Finally, expression of vimentin was also decreased by both H_2_O_2_ and GOX treatment. Although vimentin is expressed in many cell types, it is most prevalent in fibroblasts and is thought to be a partial marker of fibroblastic lineage [34]; lower expression could lead to decreased fibrosis.

To understand the mechanism by which oxidative stress mediated by GOX resulted in robust increases in cardiogenic gene expression, the expression of Notch1-related genes in GOX-treated MSCs was analyzed with a PCR array. Interestingly, Wnt11 expression was increased in GOX-treated MSCs. Wnt11 signaling has been shown to promote cardiomyogenic differentiation of human endothelial progenitor cells and mouse marrow mononuclear cells [35,36]. Moreover, Wnt signaling has been identified as a downstream target of Notch1 that regulates expression of cardiac transcription factors during mouse cardiogenesis and is essential for cardiac development [37,38]. MSCs overexpressing Wnt11 have been shown to be cardioprotective after oxidative stress in rats through increased cardiac gene expression and release of paracrine factors [39,40].

Of note, MSCs used in this study were a heterogenous mix of cells present in the adult rat bone marrow. It is unclear whether one particular lineage in the heterogenous mix is most responsible for these changes or whether Notch1 is activated in all these cell types. Although published literature suggests that all these cells express Notch [18,41], the optimal cell type must be determined.

## Conclusion

**Figure 10:**
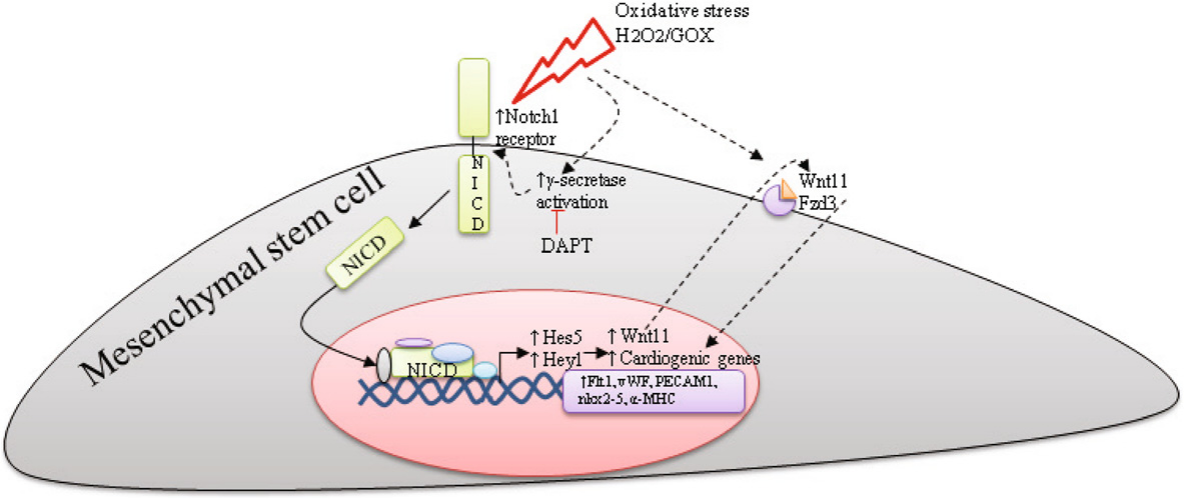
**Proposed model of H**_**2**_**O**_**2**_**/GOX-mediated induction of cardiogenic gene expression in MSCs.**

To our knowledge, this study demonstrates for the first time two important findings in the field of stem cell therapy. Oxidative stress in rat bone marrow-derived MSCs and heart-derived CPCs (a) regulates expression of selected cardiac, endothelial, and smooth muscle genes, and (b) promotes Notch1 signaling and downstream Wnt11 activation. Our current working model is depicted in Figure 10 and summarizes the findings of our study. Given that cardiogenic gene expression of two adult progenitor types (MSCs and CPCs) was induced by high levels of H_2_O_2_/GOX via Notch1 signaling, this may represent an important conserved response. As both of these cell types are in clinical trials, this study may have implications in developing adult stem/progenitor cell-based therapies.

## Abbreviations

αMHC: α-myosin heavy chain; DAPT: *N*-[*N*-(3,5-difluorophenacetyl-L-alanyl)]-*S*-phenylglycine *t*-butyl ester; Flt1: Fms-related tyrosine kinase 1; GAPDH: glyceraldehyde 3-phosphate dehydrogenase; NICD: Notch1 intracellular domain; sm22α: smooth muscle 22 alpha.

## Competing interests

The authors declare that they have no competing interests.

## Authors’ contributions

AB performed the acquisition, analysis, and interpretation of data, and drafting the manuscript; KP was involved in the acquisition and, analysis of data. PC contributed acquisition of data; YY performed conception and design; MD was involved in conception and design, critical manuscript revision, and final approval. All authors read and approved the final manuscript.

## Supplementary Material

Additional file 1 **Supplemental Data. **Description: Supplemental table and figures. Click here for file
