# Supplementary material for: Oxidative stress-induced Notch1 signaling promotes cardiogenic gene expression in mesenchymal stem cells
Source: Stem Cell Res Ther. 2013 Apr 18;4(2):43. doi: 10.1186/scrt190 (PMC3706823; doi:10.1186/scrt190)
Supplement: Additional file 1 — Supplemental Data. Description: Supplemental table and figures. [file scrt190-S1.docx]

| Gene Forward Primer Reverse Primer |
| --- |
| Flk1 5' GCCAATGAAGGGGAACTGAAGAC 3' 5' TCTGACTGCTGGTGATGCTGTC 3'  Flt1 5' CCACTTCTGTCTTGCCACACA 3' 5’ CCAACCAATTAAGACCTTCTG 3'  vWF 5' CCCACCGGATGGCTAGGTATT 3' 5' GAGGCGGATCTGTTTGAGGTT 3'  αMHC 5' AACGCCCAAGCCCACTTGAA 3' 5' CATTGGCACGGACTGCGTCA 3'  Troponin I 5' CCAGGAATCTGCAATCCCATT 3' 5' CCGCATCGCTGCTCTCA 3'  Gata4 5' ACCTGCTACAGCAGGGTTGGT 3' 5' TTCTAGCACAACTGCAAGCATGGC3'  nkx2-5 5’ CAAGTGCTCTCCTGCTTTCC 3’ 5’ GGCTTTGTCCAGCTCCACT 3’  sm α-actin 5' CCCAGATTCAGGAACAGCAT 3' 5' GTTAGCAAGGTCGGATGCTC 3'  sm22α 5’ AGCCAGTGAAGGTGCCTGAGAAC 3’ 5’ TGCCCAAAGCCATTAGAGTCCTC 3’  Notch1 5'CACCCATGACCACTACCCAGTT3' 5'CCTCGGACCAATCAGAGATGTT3'  Hey1 5'CCGCTTCGTGTTCGCCTGGT3' 5'TGCTGCCTGTGAGGGTGTCG3'  Hes5 5'ACCGCATCAACAGCAGCATT 5'AGGCTTTGCTGTGCTTCAGGT3'  Vimentin 5' CCCAGATTCAGGAACAGCAT 3' 5' CACCTGTCTCCGGTATTCGT 3'  FSP1 5' GAGGAGGCCCTGGATGTAAT 3' 5' CTTCATTGTCCCTGTTGCTG 3'  ADAM10 5’ GCCTATGTCTTCACGGACCG 3’ 5’ TGCCAGACCAAGAACACCATC 3’  ADAM17 5’ ACTCTGAGGACAGTTAACCAAACC 3’ 5’ AGTAAAAGGAGCCAATACCACAAG 3’  18S 5’ TTCCTTACCTGGTTGATCCTGCCA3’ 5’ AGCGAGCGACCAAAGGAACCATAA3’  Mef2c TaqMan MGB Rn01494046_n1  Jagged1 TaqMan MGB Rn00569647_n1  βactin TaqMan VIC MGB 4352340E |

**Additional file**

**Supplemental Data**

**Supplemental Table 1: Sequences of primers used for qRT-PCR.**

.

**Suppl Fig 1:**


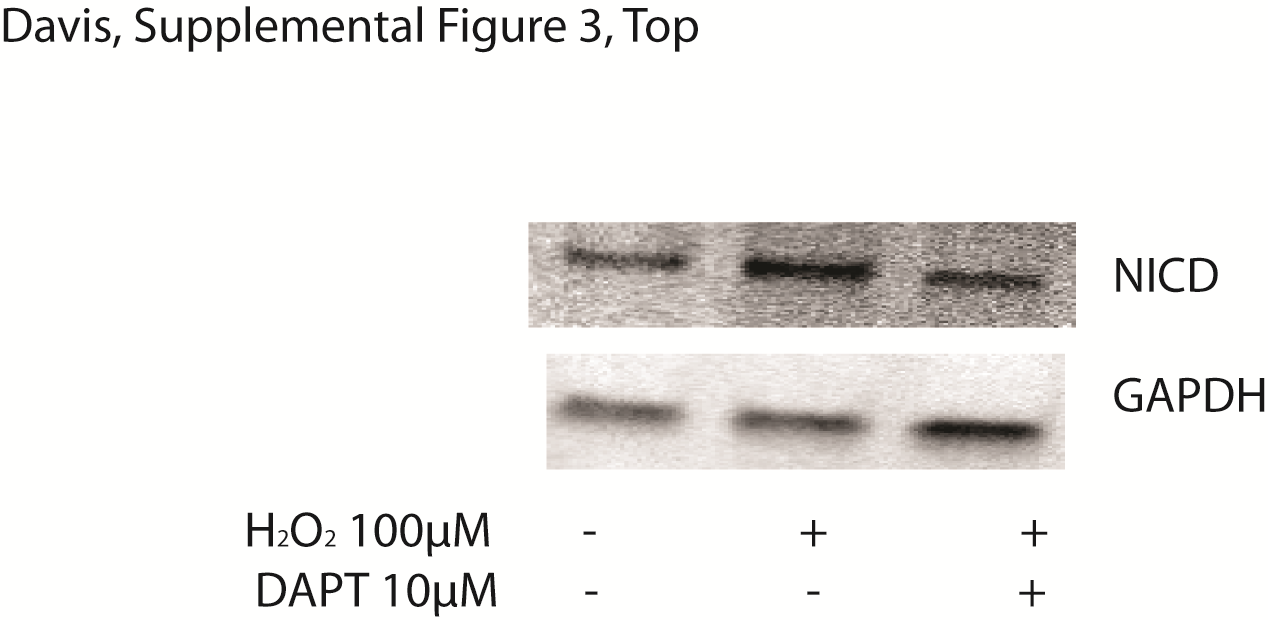


**Supplemental Figure 1: DAPT treatment decreases NICD levels following H_2_O_2_ treatment.** Representative Western blot for NICD (upper) and GAPDH (lower) in MSCs treated with vehicle, 100μM H_2_O_2_, or 100μM H_2_O_2_ + 10μM DAPT respectively.

**Suppl Fig 2:**

**Supplemental Figure 2: siNotch1 decreases Notch1 expression at the mRNA level.** (**A**) MSCs were transfected with mock siRNA labeled with Cy3 for 24h and transfection efficiency of commercially available reagents Oligofectamine, HiPerfect and Lipofectamine RNAimax was evaluated. Oligofectamine with the highest transfection efficiency was chosen for all siRNA studies. (**B**) MSCs were transfected with negative control siRNA (siNC) or siNotch1 for 48h. The mRNA expression of Notch1 is significantly decreased with siNotch1 transfection when compared to transfection reagent or siNC treated MSCs. Values are mean±SEM. * p<0.05, n≥5 by One-way ANOVA followed by Dunnett’s post test.

**Suppl Fig 3:**


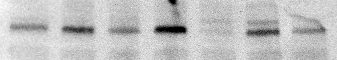

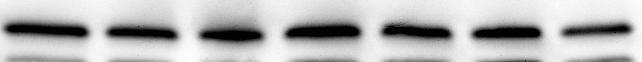


Ctrl GOX GOX+siNotch1 GOX+siNC

NICD

GAPDH

**B**

**Supplemental Figure 3: siNotch1 decreases Notch1 expression at the mRNA and protein level in presence of Glucose oxidase (GOX).** MSCs were transfected with 25nM of negative control siRNA (siNC) or siNotch1 for 48h in presence of 5mU/ml GOX. (**A**) The significant increase in mRNA expression of Notch1 upon GOX treatment is decreased to levels comparable to untreated cells by addition of siNotch1. siNC has no effect on GOX-induced Notch1 mRNA expression. Values are mean±SEM. * p<0.05, n≥5 by One-way ANOVA followed by Dunnett’s post test. (**B**) The increase in protein levels of the Notch1 Intracellular Domain (NICD) by GOX are decreased by addition of siNotch1 while levels are unaltered by siNC.

**Suppl Fig 4:**

**Supplemental Figure 4: H_2_O_2_ does not alter mRNA expression of ADAM17 and ADAM10**. MSCs were treated with 100μM H_2_O_2_ for 1 week and mRNA expression of (**A**) ADAM17 and (**B**) ADAM10 was measured by real time qPCR. Values are mean±SEM. n=10 by Student’s unpaired t-test.
